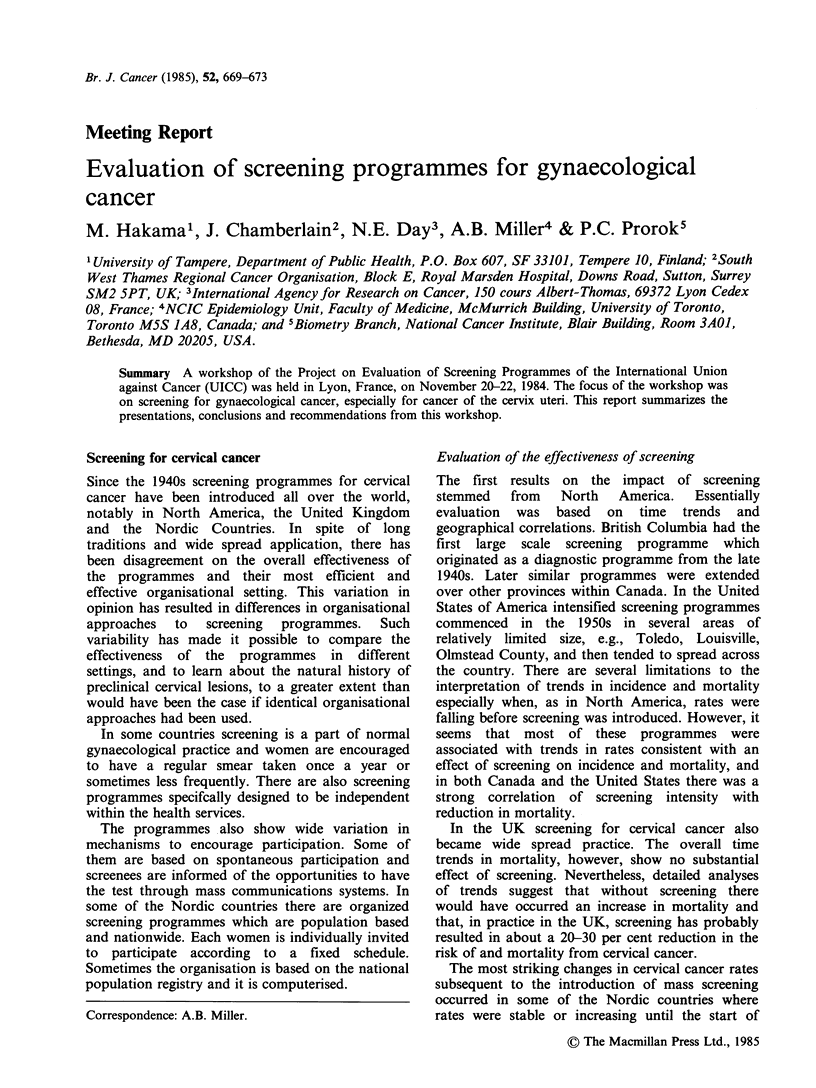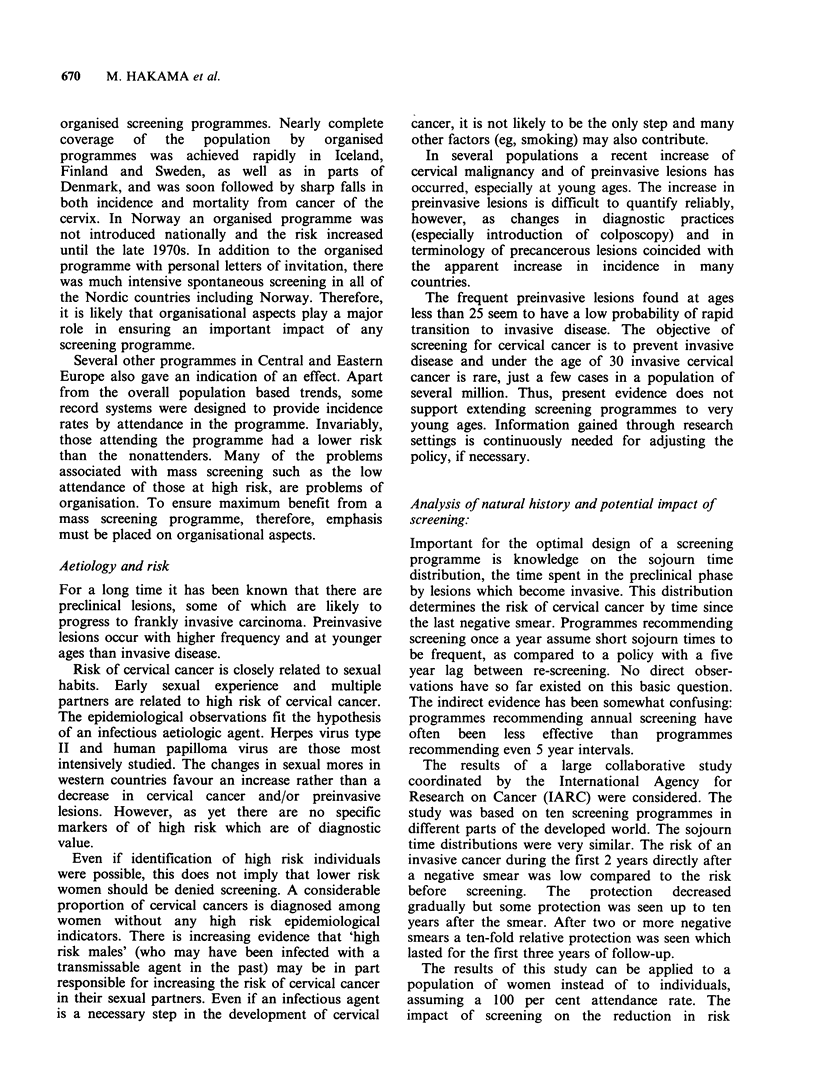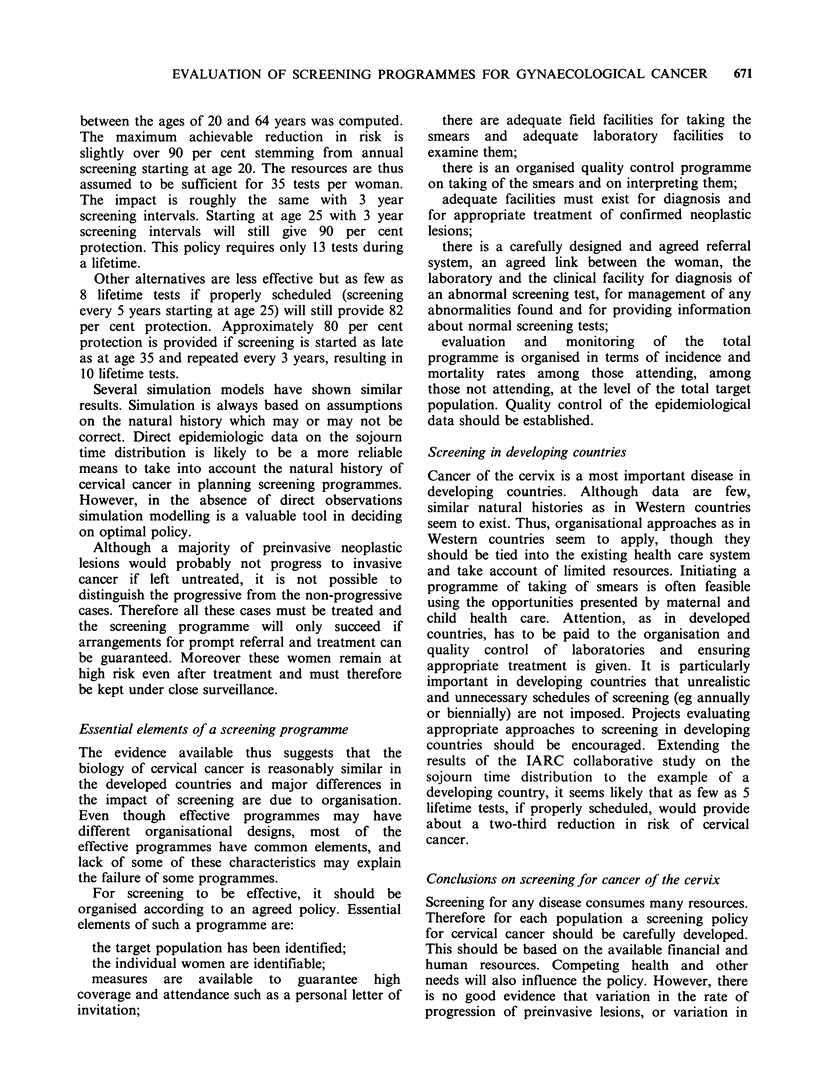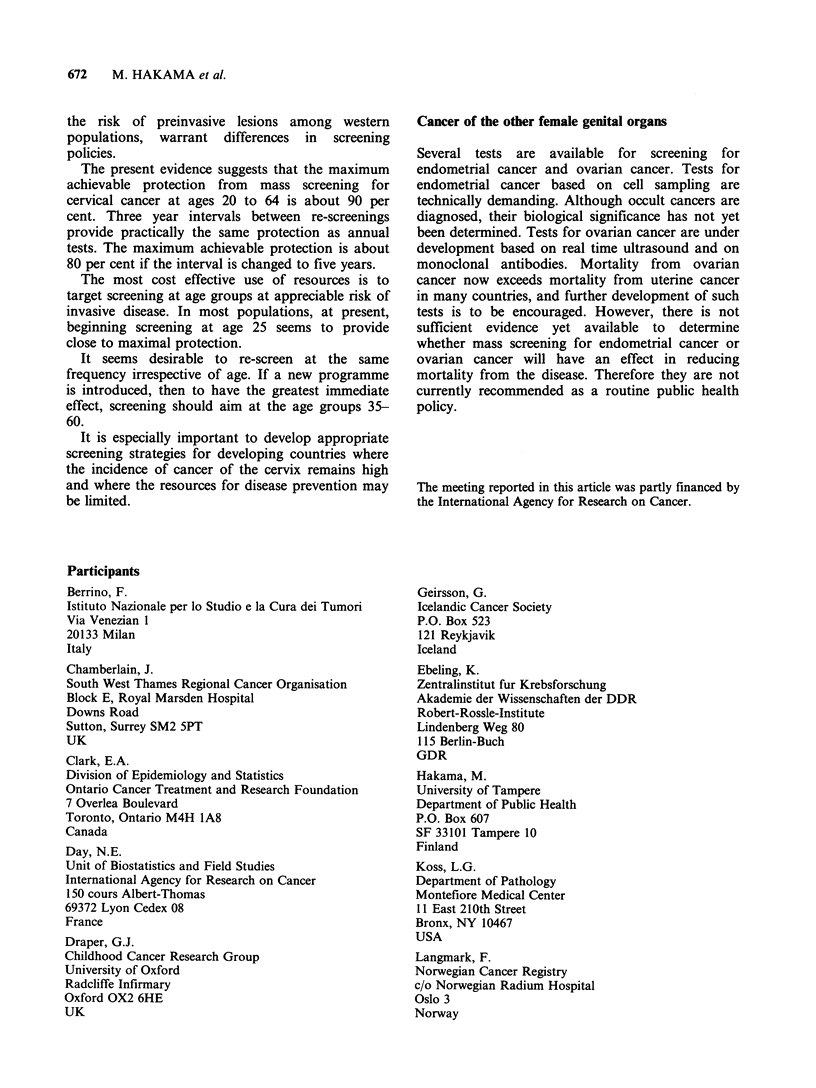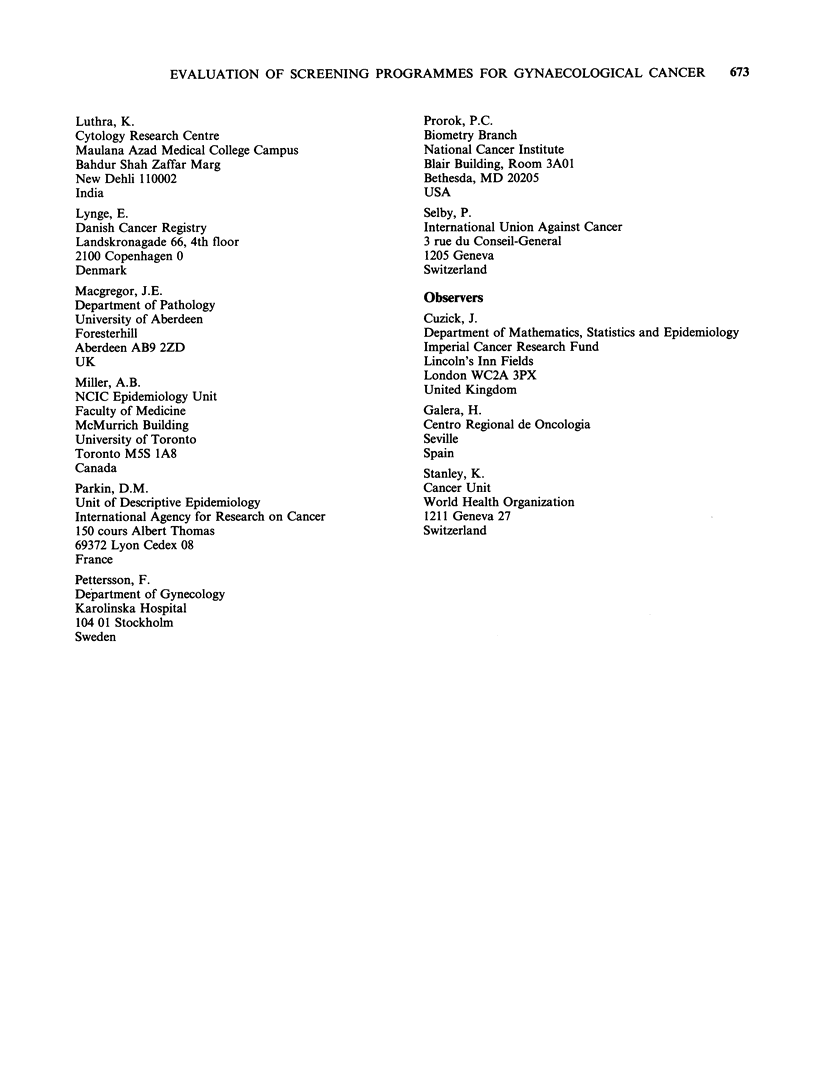# Evaluation of screening programmes for gynaecological cancer.

**DOI:** 10.1038/bjc.1985.241

**Published:** 1985-10

**Authors:** M. Hakama, J. Chamberlain, N. E. Day, A. B. Miller, P. C. Prorok

## Abstract

A workshop of the Project on Evaluation of Screening Programmes of the International Union against Cancer (UICC) was held in Lyon, France. on November 20-22, 1984. The focus of the workshop was on screening for gynaecological cancer, especially for cancer of the cervix uteri. This report summarizes the presentations, conclusions and recommendations from the workshop.


Br. J. Cancer (1985), 52, 669–673

**Meeting Report**

### Evaluation of screening programmes for gynaecological cancer

M. Hakama[1], J. Chamberlain[2], N.E. Day[3], A.B. Miller[4] & P.C. Prorok[5]

[1]*University of Tampere, Department of Public Health, P.O. Box 607, SF 33101, Tempere 10, Finland;* [2]*South West Thames Regional Cancer Organisation, Block E, Royal Marsden Hospital, Downs Road, Sutton, Surrey SM2 5PT, UK;* [3]*International Agency for Research on Cancer, 150 cours Albert-Thomas, 69372 Lyon Cedex 08, France;* [4]*NCIC Epidemiology Unit, Faculty of Medicine, McMurrich Building, University of Toronto, Toronto M5S 1A8, Canada; and* [5]*Biometry Branch, National Cancer Institute, Blair Building, Room 3A01, Bethesda, MD 20205, USA.*

**Summary** A workshop of the Project on Evaluation of Screening Programmes of the International Union against Cancer (UICC) was held in Lyon, France, on November 20–22, 1984. The focus of the workshop was on screening for gynaecological cancer, especially for cancer of the cervix uteri. This report summarizes the presentations, conclusions and recommendations from this workshop.

### Screening for cervical cancer

Since the 1940s screening programmes for cervical cancer have been introduced all over the world, notably in North America, the United Kingdom and the Nordic Countries. In spite of long traditions and wide spread application, there has been disagreement on the overall effectiveness of the programmes and their most efficient and effective organisational setting. This variation in opinion has resulted in differences in organisational approaches to screening programmes. Such variability has made it possible to compare the effectiveness of the programmes in different settings, and to learn about the natural history of preclinical cervical lesions, to a greater extent than would have been the case if identical organisational approaches had been used.

In some countries screening is a part of normal gynaecological practice and women are encouraged to have a regular smear taken once a year or sometimes less frequently. There are also screening programmes specifcally designed to be independent within the health services.

The programmes also show wide variation in mechanisms to encourage participation. Some of them are based on spontaneous participation and screenees are informed of the opportunities to have the test through mass communications systems. In some of the Nordic countries there are organized screening programmes which are population based and nationwide. Each women is individually invited to participate according to a fixed schedule. Sometimes the organisation is based on the national population registry and it is computerised.

Correspondence: A.B. Miller.

### *Evaluation of the effectiveness of screening*

The first results on the impact of screening stemmed from North America. Essentially evaluation was based on time trends and geographical correlations. British Columbia had the first large scale screening programme which originated as a diagnostic programme from the late 1940s. Later similar programmes were extended over other provinces within Canada. In the United States of America intensified screening programmes commenced in the 1950s in several areas of relatively limited size, e.g., Toledo, Louisville, Olmstead County, and then tended to spread across the country. There are several limitations to the interpretation of trends in incidence and mortality especially when, as in North America, rates were falling before screening was introduced. However, it seems that most of these programmes were associated with trends in rates consistent with an effect of screening on incidence and mortality, and in both Canada and the United States there was a strong correlation of screening intensity with reduction in mortality.

In the UK screening for cervical cancer also became wide spread practice. The overall time trends in mortality, however, show no substantial effect of screening. Nevertheless, detailed analyses of trends suggest that without screening there would have occurred an increase in mortality and that, in practice in the UK, screening has probably resulted in about a 20–30 per cent reduction in the risk of and mortality from cervical cancer.

The most striking changes in cervical cancer rates subsequent to the introduction of mass screening occurred in some of the Nordic countries where rates were stable or increasing until the start of

© The Macmillan Press Ltd., 1985

organised screening programmes. Nearly complete coverage of the population by organised programmes was achieved rapidly in Iceland, Finland and Sweden, as well as in parts of Denmark, and was soon followed by sharp falls in both incidence and mortality from cancer of the cervix. In Norway an organised programme was not introduced nationally and the risk increased until the late 1970s. In addition to the organised programme with personal letters of invitation, there was much intensive spontaneous screening in all of the Nordic countries including Norway. Therefore, it is likely that organisational aspects play a major role in ensuring an important impact of any screening programme.

Several other programmes in Central and Eastern Europe also gave an indication of an effect. Apart from the overall population based trends, some record systems were designed to provide incidence rates by attendance in the programme. Invariably, those attending the programme had a lower risk than the nonattenders. Many of the problems associated with mass screening such as the low attendance of those at high risk, are problems of organisation. To ensure maximum benefit from a mass screening programme, therefore, emphasis must be placed on organisational aspects.

*Aetiology and risk*

For a long time it has been known that there are preclinical lesions, some of which are likely to progress to frankly invasive carcinoma. Preinvasive lesions occur with higher frequency and at younger ages than invasive disease.

Risk of cervical cancer is closely related to sexual habits. Early sexual experience and multiple partners are related to high risk of cervical cancer. The epidemiological observations fit the hypothesis of an infectious aetiologic agent. Herpes virus type II and human papilloma virus are those most intensively studied. The changes in sexual mores in western countries favour an increase rather than a decrease in cervical cancer and/or preinvasive lesions. However, as yet there are no specific markers of of high risk which are of diagnostic value.

Even if identification of high risk individuals were possible, this does not imply that lower risk women should be denied screening. A considerable proportion of cervical cancers is diagnosed among women without any high risk epidemiological indicators. There is increasing evidence that 'high risk males' (who may have been infected with a transmissable agent in the past) may be in part responsible for increasing the risk of cervical cancer in their sexual partners. Even if an infectious agent is a necessary step in the development of cervical cancer, it is not likely to be the only step and many other factors (eg, smoking) may also contribute.

In several populations a recent increase of cervical malignancy and of preinvasive lesions has occurred, especially at young ages. The increase in preinvasive lesions is difficult to quantify reliably, however, as changes in diagnostic practices (especially introduction of colposcopy) and in terminology of precancerous lesions coincided with the apparent increase in incidence in many countries.

The frequent preinvasive lesions found at ages less than 25 seem to have a low probability of rapid transition to invasive disease. The objective of screening for cervical cancer is to prevent invasive disease and under the age of 30 invasive cervical cancer is rare, just a few cases in a population of several million. Thus, present evidence does not support extending screening programmes to very young ages. Information gained through research settings is continuously needed for adjusting the policy, if necessary.

*Analysis of natural history and potential impact of screening:*

Important for the optimal design of a screening programme is knowledge on the sojourn time distribution, the time spent in the preclinical phase by lesions which become invasive. This distribution determines the risk of cervical cancer by time since the last negative smear. Programmes recommending screening once a year assume short sojourn times to be frequent, as compared to a policy with a five year lag between re-screening. No direct observations have so far existed on this basic question. The indirect evidence has been somewhat confusing: programmes recommending annual screening have often been less effective than programmes recommending even 5 year intervals.

The results of a large collaborative study coordinated by the International Agency for Research on Cancer (IARC) were considered. The study was based on ten screening programmes in different parts of the developed world. The sojourn time distributions were very similar. The risk of an invasive cancer during the first 2 years directly after a negative smear was low compared to the risk before screening. The protection decreased gradually but some protection was seen up to ten years after the smear. After two or more negative smears a ten-fold relative protection was seen which lasted for the first three years of follow-up.

The results of this study can be applied to a population of women instead of to individuals, assuming a 100 per cent attendance rate. The impact of screening on the reduction in risk between the ages of 20 and 64 years was computed. The maximum achievable reduction in risk is slightly over 90 per cent stemming from annual screening starting at age 20. The resources are thus assumed to be sufficient for 35 tests per woman. The impact is roughly the same with 3 year screening intervals. Starting at age 25 with 3 year screening intervals will still give 90 per cent protection. This policy requires only 13 tests during a lifetime.

Other alternatives are less effective but as few as 8 lifetime tests if properly scheduled (screening every 5 years starting at age 25) will still provide 82 per cent protection. Approximately 80 per cent protection is provided if screening is started as late as at age 35 and repeated every 3 years, resulting in 10 lifetime tests.

Several simulation models have shown similar results. Simulation is always based on assumptions on the natural history which may or may not be correct. Direct epidemiologic data on the sojourn time distribution is likely to be a more reliable means to take into account the natural history of cervical cancer in planning screening programmes. However, in the absence of direct observations simulation modelling is a valuable tool in deciding on optimal policy.

Although a majority of preinvasive neoplastic lesions would probably not progress to invasive cancer if left untreated, it is not possible to distinguish the progressive from the non-progressive cases. Therefore all these cases must be treated and the screening programme will only succeed if arrangements for prompt referral and treatment can be guaranteed. Moreover these women remain at high risk even after treatment and must therefore be kept under close surveillance.

### *Essential elements of a screening programme*

The evidence available thus suggests that the biology of cervical cancer is reasonably similar in the developed countries and major differences in the impact of screening are due to organisation. Even though effective programmes may have different organisational designs, most of the effective programmes have common elements, and lack of some of these characteristics may explain the failure of some programmes.

For screening to be effective, it should be organised according to an agreed policy. Essential elements of such a programme are:

the target population has been identified; the individual women are identifiable; measures are available to guarantee high coverage and attendance such as a personal letter of invitation; there are adequate field facilities for taking the smears and adequate laboratory facilities to examine them; there is an organised quality control programme on taking of the smears and on interpreting them; adequate facilities must exist for diagnosis and for appropriate treatment of confirmed neoplastic lesions; there is a carefully designed and agreed referral system, an agreed link between the woman, the laboratory and the clinical facility for diagnosis of an abnormal screening test, for management of any abnormalities found and for providing information about normal screening tests; evaluation and monitoring of the total programme is organised in terms of incidence and mortality rates among those attending, among those not attending, at the level of the total target population. Quality control of the epidemiological data should be established.

### *Screening in developing countries*

Cancer of the cervix is a most important disease in developing countries. Although data are few, similar natural histories as in Western countries seem to exist. Thus, organisational approaches as in Western countries seem to apply, though they should be tied into the existing health care system and take account of limited resources. Initiating a programme of taking of smears is often feasible using the opportunities presented by maternal and child health care. Attention, as in developed countries, has to be paid to the organisation and quality control of laboratories and ensuring appropriate treatment is given. It is particularly important in developing countries that unrealistic and unnecessary schedules of screening (eg annually or biennially) are not imposed. Projects evaluating appropriate approaches to screening in developing countries should be encouraged. Extending the results of the IARC collaborative study on the sojourn time distribution to the example of a developing country, it seems likely that as few as 5 lifetime tests, if properly scheduled, would provide about a two-third reduction in risk of cervical cancer.

### *Conclusions on screening for cancer of the cervix*

Screening for any disease consumes many resources. Therefore for each population a screening policy for cervical cancer should be carefully developed. This should be based on the available financial and human resources. Competing health and other needs will also influence the policy. However, there is no good evidence that variation in the rate of progression of preinvasive lesions, or variation in the risk of preinvasive lesions among western populations, warrant differences in screening policies.

The present evidence suggests that the maximum achievable protection from mass screening for cervical cancer at ages 20 to 64 is about 90 per cent. Three year intervals between re-screenings provide practically the same protection as annual tests. The maximum achievable protection is about 80 per cent if the interval is changed to five years.

The most cost effective use of resources is to target screening at age groups at appreciable risk of invasive disease. In most populations, at present, beginning screening at age 25 seems to provide close to maximal protection.

It seems desirable to re-screen at the same frequency irrespective of age. If a new programme is introduced, then to have the greatest immediate effect, screening should aim at the age groups 35–60.

It is especially important to develop appropriate screening strategies for developing countries where the incidence of cancer of the cervix remains high and where the resources for disease prevention may be limited.

**Cancer of the other female genital organs**

Several tests are available for screening for endometrial cancer and ovarian cancer. Tests for endometrial cancer based on cell sampling are technically demanding. Although occult cancers are diagnosed, their biological significance has not yet been determined. Tests for ovarian cancer are under development based on real time ultrasound and on monoclonal antibodies. Mortality from ovarian cancer now exceeds mortality from uterine cancer in many countries, and further development of such tests is to be encouraged. However, there is not sufficient evidence yet available to determine whether mass screening for endometrial cancer or ovarian cancer will have an effect in reducing mortality from the disease. Therefore they are not currently recommended as a routine public health policy.

The meeting reported in this article was partly financed by the International Agency for Research on Cancer.

**Participants**

Berrino, F.
Istituto Nazionale per lo Studio e la Cura dei Tumori
Via Venezian 1
20133 Milan
Italy

Chamberlain, J.
South West Thames Regional Cancer Organisation
Block E, Royal Marsden Hospital
Downs Road
Sutton, Surrey SM2 5PT
UK

Clark, E.A.
Division of Epidemiology and Statistics
Ontario Cancer Treatment and Research Foundation
7 Overlea Boulevard
Toronto, Ontario M4H 1A8
Canada

Day, N.E.
Unit of Biostatistics and Field Studies
International Agency for Research on Cancer
150 cours Albert-Thomas
69372 Lyon Cedex 08
France

Draper, G.J.
Childhood Cancer Research Group
University of Oxford
Radcliffe Infirmary
Oxford OX2 6HE
UK

Geirsson, G.
Icelandic Cancer Society
P.O. Box 523
121 Reykjavik
Iceland

Ebeling, K.
Zentralinstitut fur Krebsforschung
Akademie der Wissenschaften der DDR
Robert-Rossle-Institute
Lindenberg Weg 80
115 Berlin-Buch
GDR

Hakama, M.
University of Tampere
Department of Public Health
P.O. Box 607
SF 33101 Tampere 10
Finland

Koss, L.G.
Department of Pathology
Montefiore Medical Center
11 East 210th Street
Bronx, NY 10467
USA

Langmark, F.
Norwegian Cancer Registry
c/o Norwegian Radium Hospital
Oslo 3
Norway

Luthra, K.
Cytology Research Centre
Maulana Azad Medical College Campus
Bahdur Shah Zaffar Marg
New Dehli 110002
India

Lynge, E.
Danish Cancer Registry
Landskronagade 66, 4th floor
2100 Copenhagen 0
Denmark

Macgregor, J.E.
Department of Pathology
University of Aberdeen
Foresterhill
Aberdeen AB9 2ZD
UK

Miller, A.B.
NCIC Epidemiology Unit
Faculty of Medicine
McMurrich Building
University of Toronto
Toronto M5S 1A8
Canada

Parkin, D.M.
Unit of Descriptive Epidemiology
International Agency for Research on Cancer
150 cours Albert Thomas
69372 Lyon Cedex 08
France

Pettersson, F.
Department of Gynecology
Karolinska Hospital
104 01 Stockholm
Sweden

Prorok, P.C.
Biometry Branch
National Cancer Institute
Blair Building, Room 3A01
Bethesda, MD 20205
USA

Selby, P.
International Union Against Cancer
3 rue du Conseil-General
1205 Geneva
Switzerland

### Observers

Cuzick, J.
Department of Mathematics, Statistics and Epidemiology
Imperial Cancer Research Fund
Lincoln's Inn Fields
London WC2A 3PX
United Kingdom

Galera, H.
Centro Regional de Oncologia
Seville
Spain

Stanley, K.
Cancer Unit
World Health Organization
1211 Geneva 27
Switzerland